# Supplementary figures and images for: Analysis of Stop-Gain and Frameshift Variants in Human Innate Immunity Genes
Source: PLoS Comput Biol. 2014 Jul 24;10(7):e1003757. doi: 10.1371/journal.pcbi.1003757 (PMC4110073; doi:10.1371/journal.pcbi.1003757)

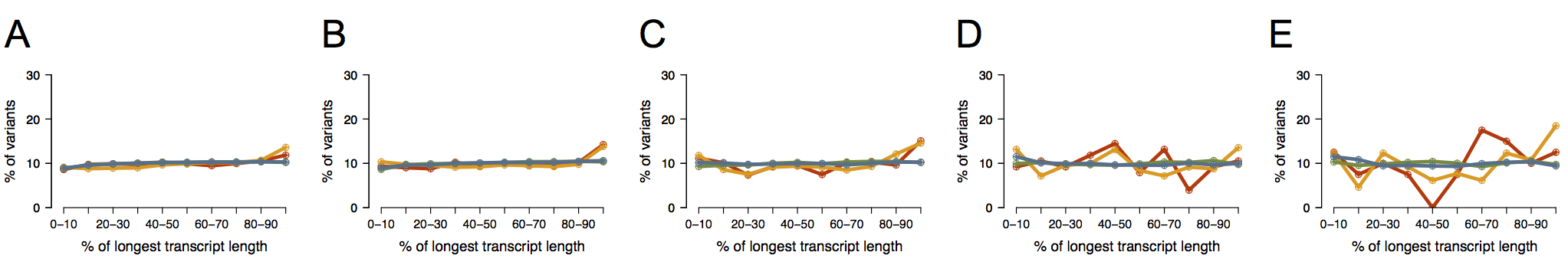

Supplement: Figure S1 — Distribution of variants along the gene sequence. The distribution is shown for synonymous (green), missense (blue), stop-gain (red) and frameshift (orange) variants binned by minor allele frequency (MAF) intervals: Singletons (panel A), MAF<0.001 (Panel B), MAF [0.001–0.01) (Panel C), MAF [0.01–0.05) (Panel D) and MAF>0.05 (Panel E). Numbers of variants in each category are reported in Table S1. Data were combined across the sequence using intervals of 10%. The longest transcript for each gene was used as the reference sequence length. (TIF) [file pcbi.1003757.s001.tif]

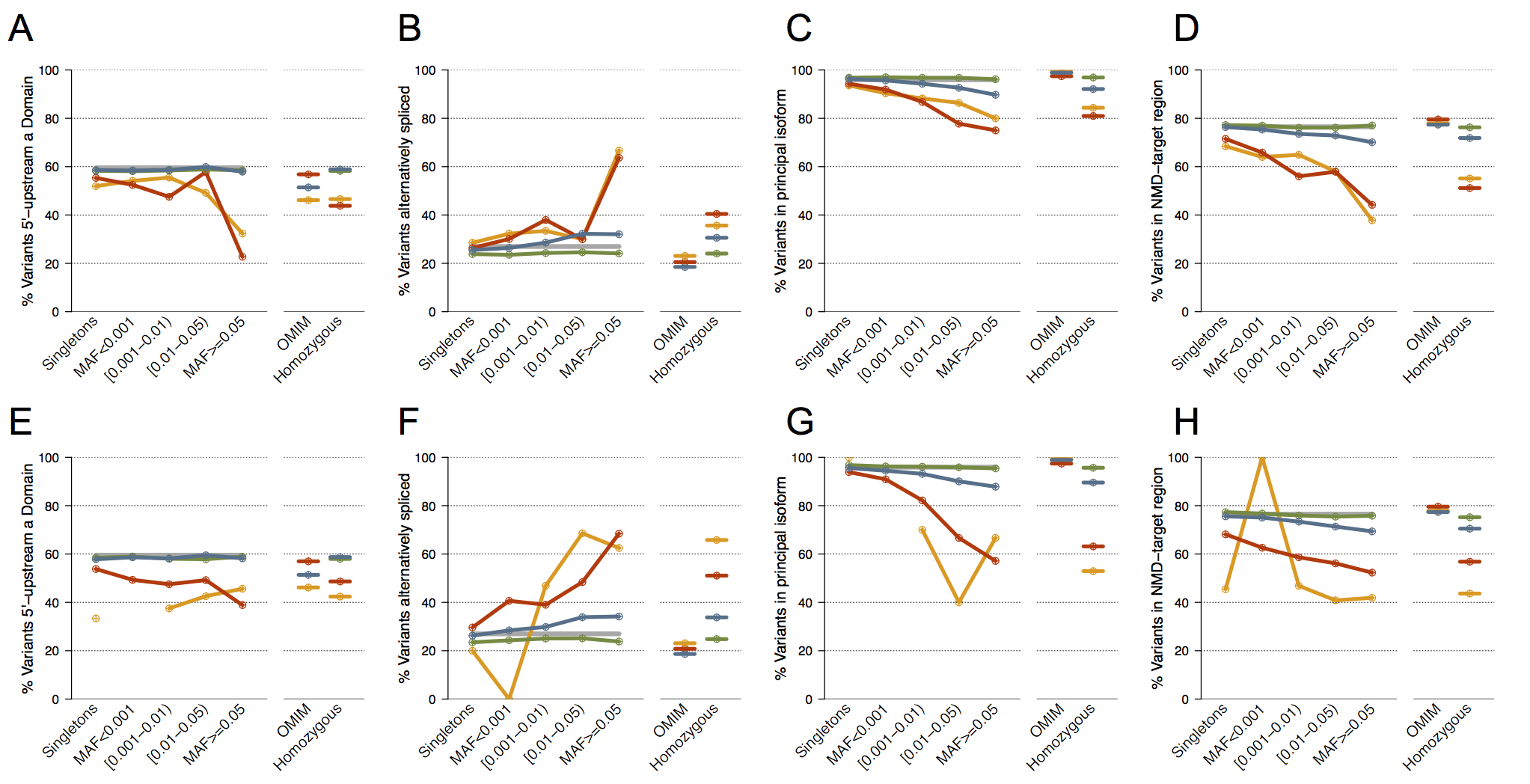

Supplement: Figure S2 — Distribution of variants according to sequence features and allele frequency represented separately for the ESP and the 1000 Genomes datasets. The percentage of variants upstream of a functional domain (Panels A and E), in alternatively spliced sites (Panel B and F), in the principal isoform (panel C and G) and in regions targeted by NMD (Panel D and H). Panels A, B, C and D correspond to variants in the ESP dataset, and panels E, F, G and H to the 1000 Genomes dataset. The distribution is shown for synonymous (green), missense (blue), stop-gains (red) and frameshift (orange) variants according to minor allele frequency (MAF) intervals, where singletons (variants detected only in one individual) are represented separately. The pattern of OMIM disease variants and homozygous variants for each feature is shown. The corresponding coding genome background (measured as the percentage of nucleotides displaying the feature) is shown as a grey line (partly hidden by the distribution of synonymous variants in some panels). The y-axis represents the percentage of variants for the categories represented in the x-axis. Logistic regression was used to model the relationship between observing a given sequence feature in a given type of variant as a function of the logarithm of the minor allele frequency (MAF). In the ESP dataset, the odds ratio estimates for stop-gain variants were significantly different from those of synonymous variants in all panels (p-values<1e-04, heterogeneity test [1]; for frameshifts, in panels B, C and D (p-values<5e-02). In the 1000G dataset, the odds ratio estimates for stop-gain variants were significantly different from those of synonymous variants in all panels (p-values≪5e-02, heterogeneity test [1]; for frameshifts, in panel F (p-value<5e-02). Distribution for frameshift variants from the 1000 Genomes dataset is noisy due to small sample size (Table S1). (TIF) [file pcbi.1003757.s002.tif]

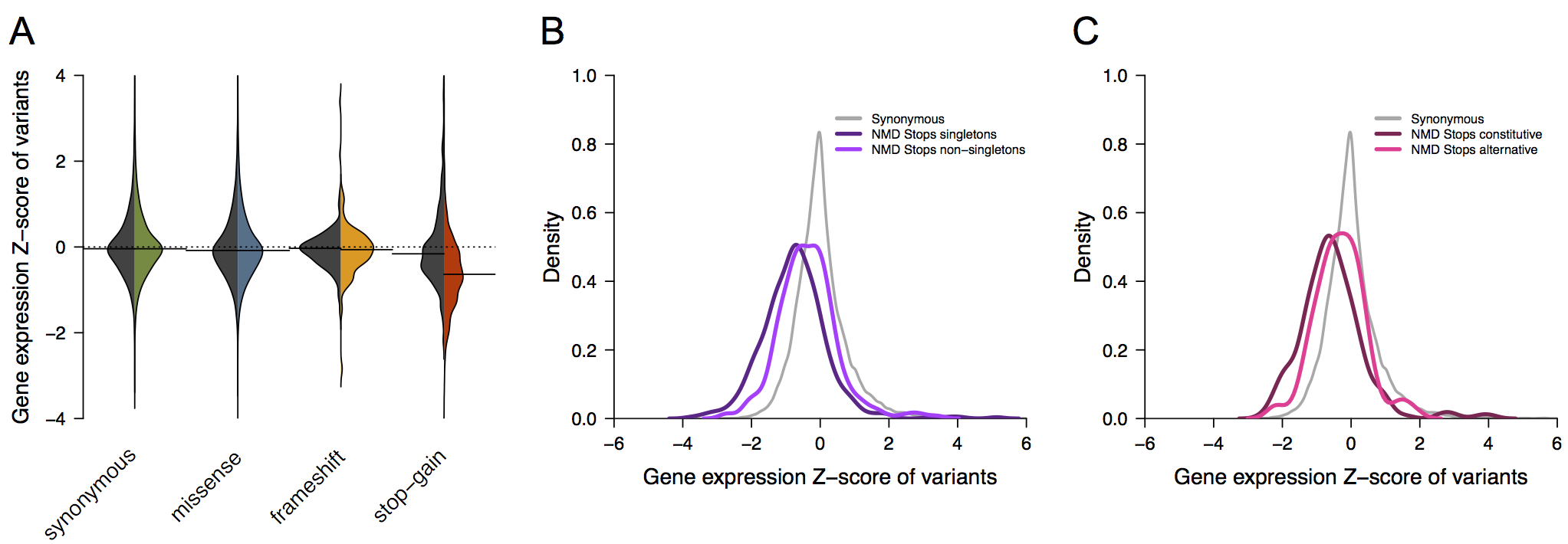

Supplement: Figure S3 — Association of NMD-target variants with gene expression using standard RPKM normalization. Results in Figure 2 are reproduced here using standard RPKM normalized expression values from Lappalainen et al. [2]. Panel A shows the distribution of average expression z-scores for genes from individuals carrying different types of variants (synonymous, missense, frameshift and stop-gain). The black half represents the distribution of variants outside the NMD-target region and the colored half for those within the NMD-target region. As in Figure 2 , statistically significant differences were observed for stop-gain variants predicted to trigger NMD (n = 756) compared to synonymous variants (one-sided Wilcoxon rank-sum test p-value<2.2e-16). Panel B shows the distribution of average expression z-scores described in panel A for synonymous (grey) and stop-gain (dark and light purple) variants within the NMD-target region. The distribution of NMD-target stop-gains is represented separately for singletons (dark purple, n = 488) and non-singletons (n = 268). Distributions are statistically different (one-sided Wilcoxon rank-sum test = 4.4e-10). Panel C shows the distribution of average expression z-scores described in panel A for synonymous (grey) and stop-gain (dark and light pink) variants within the NMD-target region of genes with multiple isoforms described in CCDS. The distribution of NMD-target stop-gain is represented separately for those affecting all isoforms (dark pink, n = 216) and those affecting only a fraction of isoforms (light pink, n = 85). As in Figure 2 , distributions are statistically different (one-sided Wilcoxon rank-sum test = 1.5e-03). (TIF) [file pcbi.1003757.s003.tif]

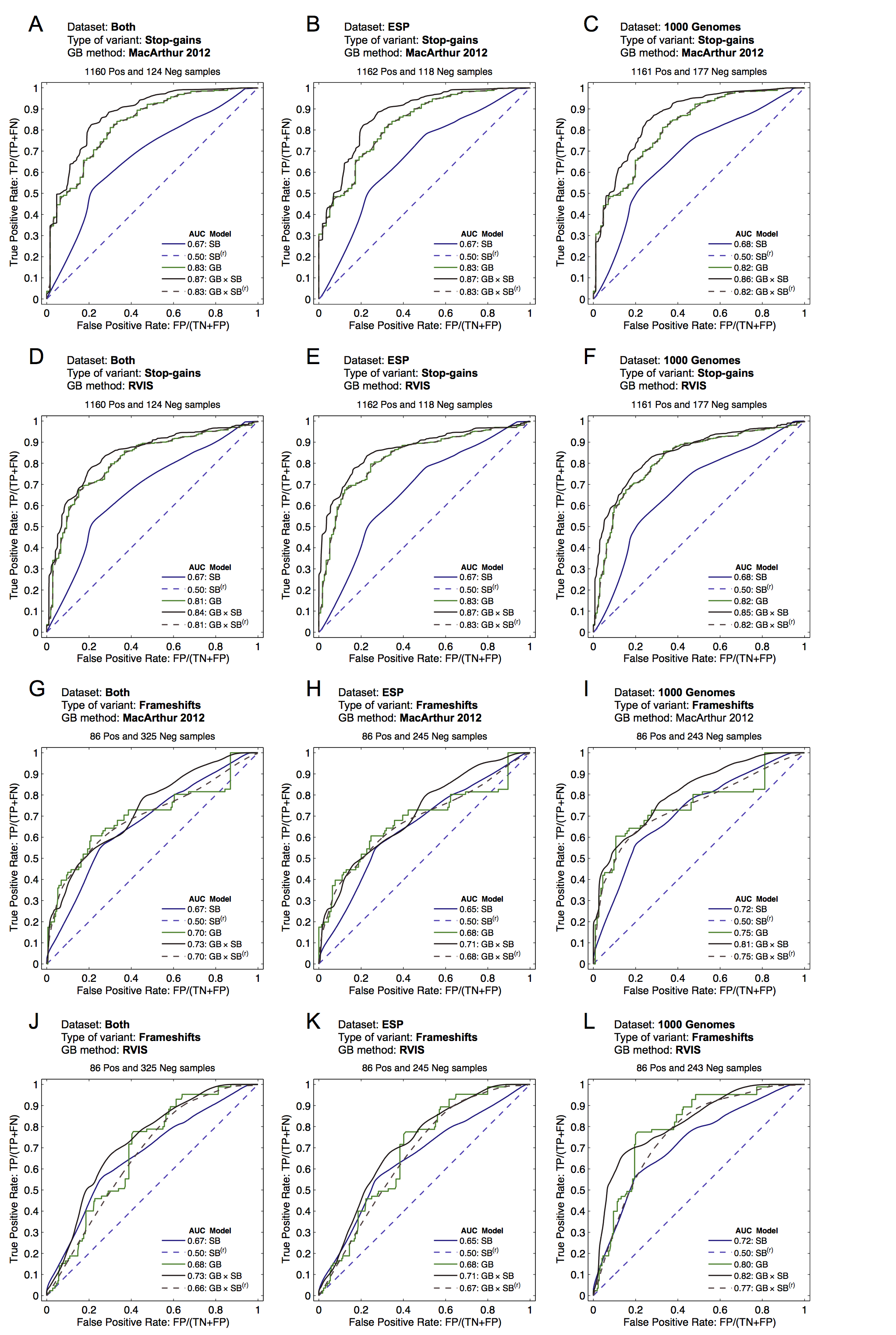

Supplement: Figure S4 — Receiver operating characteristic of the performance of pathogenicity scores for stop and frameshift variants. Shown are the ROC curves corresponding to the sequence-based classifier (SB) developed in this work, a gene-based scores (GB) (Panels A–F: MacArthur 2012 [3]; Panels G–L: RVIS [4]), and the joint score combining the sequence-based and a gene-based score (SB×GB). Dashed curves correspond to a randomization test in which rows in sequence features are shuffled column-wise (denoted by SB(r) and GBxSB(r)). Classification power was evaluated on a set of pathogenic variants found in OMIM database (referred in the figure as Positives (Pos), and common variants not known to be pathogenic (referred in the figure as Negatives (Neg). Total number of Positive and Negative variants used is indicated above each panel. Panels A–C and G–I represent stop-gain variants while Panels D–F and J–L represent frameshif variants. Results are shown for both the ESP and 1000 Genomes datasets considered together (Panels A, D, G, J) or separately (Panels B, E, H, K for the ESP dataset and panels C, F, I, L for the 1000 Genomes dataset). Number of pathogenic and common variants used for benchmarking is shown on top of each panel. AUC values of ROC curves for each model are indicated. Incorporating sequence features led to an increased area under the ROC curve in all evaluated settings ( Figure 3B ). (TIF) [file pcbi.1003757.s004.tif]

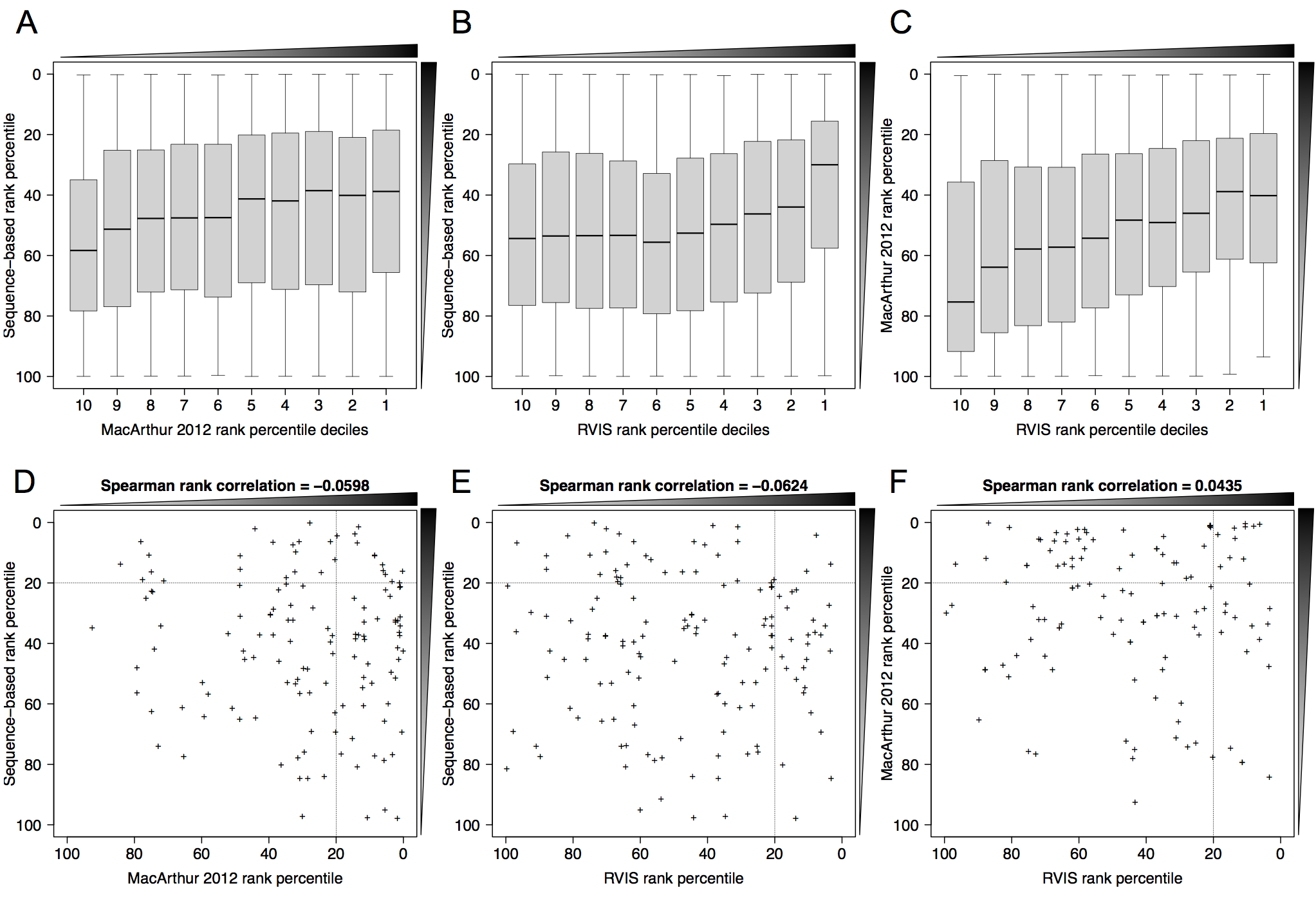

Supplement: Figure S5 — Correlation between sequence-based scores and gene-based scores for truncating variants. Figure shows the correlation between the sequence-based pathogenicity score developed in this work and two gene-based pathogenicity scores (Panels A and D: MacArthur 2012 [3]; Panels B and E: RVIS [4]). Correlation between the two gene-based scores is shown in Panels C and F. Panels A–C represent values for 17645 stop-gain variants reported by the ESP and the 1000 Genomes datasets (panels A–C). Panels D–F represent values for 155 disease stop-gain variants annotated as pathogenic by OMIM and reported by the ESP and the 1000 Genomes datasets (we note that OMIM variants used here were not considered for learning in the Bayesian classification; see Methods). Upper Panels A–C display the distribution of the score on the y-axis in the form of boxplots conditioned to decile bins of the score on the x-axis. Lower Panels D–E represent the values for each individual OMIM variant (depicted with cross marks). For comparison across scores, they are represented as rank percentiles, where the value of a given variant accounts for the percentage of all stop-variants that had a score more pathogenic than the variant. Therefore, a rank percentile of “0” indicates a variant with the highest predicted probability of being pathogenic while a rank percentile of “100” indicates a variant with the lowest predicted severity. Grey triangles beside the panels represent the direction of increasing pathogenicity for the corresponding variable. Lines in Panels D–F divide variants in four regimes according to their belonging to the top 20% pathogenicity ranking of the corresponding scores, the top-right regime being the one where both scores agreed. Spearman rank correlation tests yielded significant p-values in panels A–C (p-value<2.2e-16). Spearman correlations were <0.13 (panels A and B; [0.107,0.125] and [0.115,0.123] 95% CI from 10,000 bootstrap samples, respectively), <0.24 (panel C; [0.224,0.241] 95% [file pcbi.1003757.s005.tif]

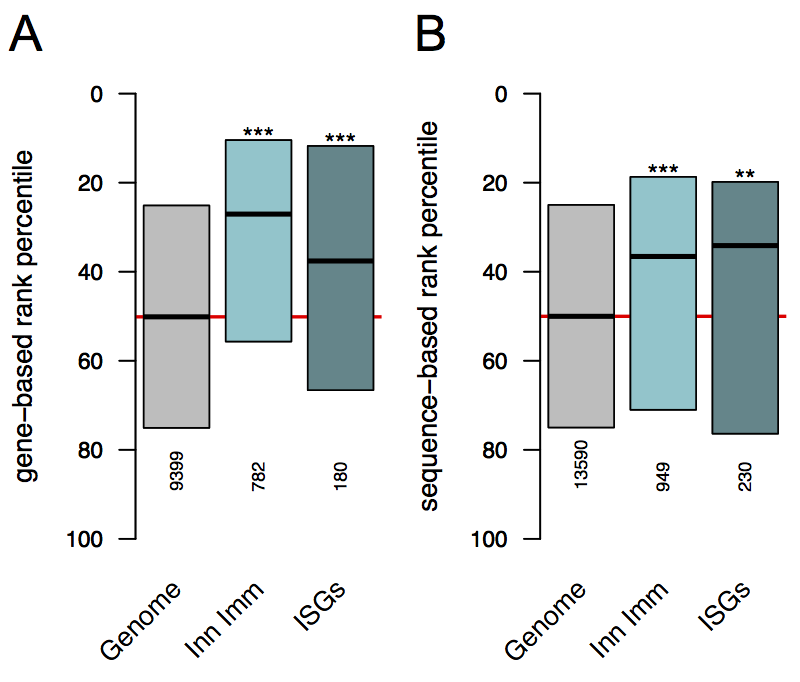

Supplement: Figure S6 — Pathogenicity score distributions for rare frameshift variants in innate immunity genes. Rank percentile distributions of pathogenicity scores for rare frameshift variants (MAF<1%) are shown in different sets of genes: protein coding genome background (grey, “Genome”), innate immunity genes (light turquoise, “Inn Imm”) and their subset of interferon stimulated genes (dark turquoise, “ISGs”). In contrast with Figure 7 , the same categories for OMIM disease frameshifts are not shown due to low number or absence of variants. All variants are reported in ESP and 1000 Genomes Projects. Variants with the highest probability of being pathogenic have rank percentiles closer to zero (top of the panels). Panel A represents precomputed gene-based pathogenicity scores from [3]. Panel B represents sequence-based pathogenicity scores, i.e. posterior probabilities using the features described in the present work (see main text). Each box spans between 1st and 3rd quantile, and the median is denoted by a bold line in the middle. Total number of variants within each distribution is indicated. Differences in number of variants in equivalent categories between panel A and B originate from unavailability of the gene-based scores for some genes. Statistical differences against the genome reference (one-sided Wilcoxon rank sum tests) are indicated with asterisks according to Bonferroni corrected p-values: <5e-02 (*), <5e-03 (**) and <5e-04 (**). The genome-wide median is denoted by a red line. Spearman correlation between the sequenced-based and gene-based pathogenicity scores was below 0.13 in all sets of genes analyzed. (TIF) [file pcbi.1003757.s006.tif]

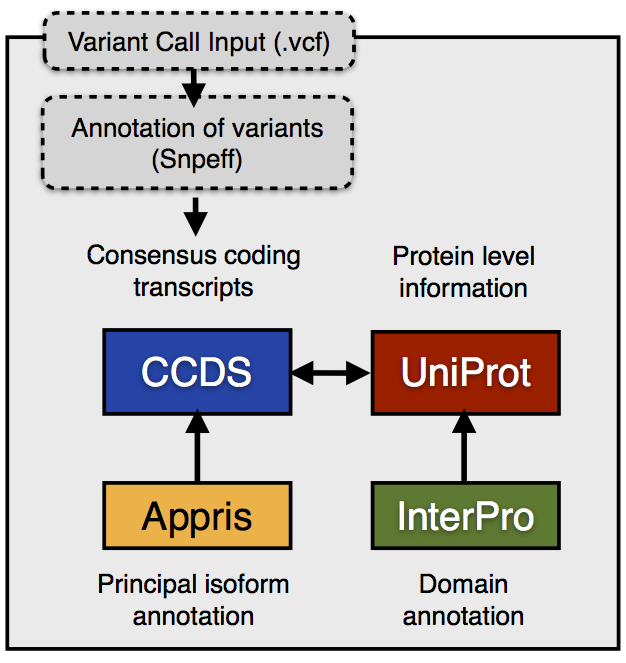

Supplement: Figure S7 — Pipeline implemented to annotate genetic variants in reference human transcripts and protein sequences. Figure depicts the schematic pipeline followed for the annotation of variants (see Methods). Analysis was restricted to variants affecting autosomal protein coding genes and transcripts annotated by the Consensus CDS (CCDS) project ([5]. Annotation of principal isoforms used APPRIS system ([6]. Transcript-based information was related to protein-based information through UniProt [7]. InterPro database ([8] was used to retrieve protein domain information. (TIF) [file pcbi.1003757.s007.tif]
