# Supplementary material for: Analysis of Stop-Gain and Frameshift Variants in Human Innate Immunity Genes
Source: PLoS Comput Biol. 2014 Jul 24;10(7):e1003757. doi: 10.1371/journal.pcbi.1003757 (PMC4110073; doi:10.1371/journal.pcbi.1003757)
Supplement: Text S1 — References in Supplementary Information legends. (DOCX) [file pcbi.1003757.s012.docx]

**Text S1. References in Supplementary Information legends.**

1. Randall JC, Winkler TW, Kutalik Z, Berndt SI, Jackson AU, et al. (2013) Sex-stratified genome-wide association studies including 270,000 individuals show sexual dimorphism in genetic loci for anthropometric traits. PLoS Genet 9: e1003500.

2. Lappalainen T, Sammeth M, Friedlander MR, t Hoen PA, Monlong J, et al. (2013) Transcriptome and genome sequencing uncovers functional variation in humans. Nature 501: 506-511.

3. MacArthur DG, Balasubramanian S, Frankish A, Huang N, Morris J, et al. (2012) A systematic survey of loss-of-function variants in human protein-coding genes. Science 335: 823-828.

4. Petrovski S, Wang Q, Heinzen EL, Allen AS, Goldstein DB (2013) Genic intolerance to functional variation and the interpretation of personal genomes. PLoS Genet 9: e1003709.

5. Pruitt KD, Harrow J, Harte RA, Wallin C, Diekhans M, et al. (2009) The consensus coding sequence (CCDS) project: Identifying a common protein-coding gene set for the human and mouse genomes. Genome Res 19: 1316-1323.

6. Rodriguez JM, Maietta P, Ezkurdia I, Pietrelli A, Wesselink JJ, et al. (2013) APPRIS: annotation of principal and alternative splice isoforms. Nucleic Acids Res 41: D110-117.

7. UniProt C (2013) Update on activities at the Universal Protein Resource (UniProt) in 2013. Nucleic Acids Res 41: D43-47.

8. Hunter S, Jones P, Mitchell A, Apweiler R, Attwood TK, et al. (2012) InterPro in 2011: new developments in the family and domain prediction database. Nucleic Acids Res 40: D306-312.

9. Schoggins JW, Wilson SJ, Panis M, Murphy MY, Jones CT, et al. (2011) A diverse range of gene products are effectors of the type I interferon antiviral response. Nature 472: 481-485.

10. Schoggins JW, Macduff DA, Imanaka N, Gainey MD, Shrestha B, et al. (2013) Pan-viral specificity of IFN-induced genes reveals new roles for cGAS in innate immunity. Nature.
